# Unveiling the hidden genetic diversity and chloroplast type of marine benthic ciliate *Mesodinium* species

**DOI:** 10.1038/s41598-019-50659-2

**Published:** 2019-10-01

**Authors:** Miran Kim, Myung Gil Park

**Affiliations:** 1 Research Institute for Basic Science, Chonnam National University, Gwangju, 61186 Republic of Korea; 2 LOHABE, Department of Oceanography, Chonnam National University, Gwangju, 61186 Republic of Korea

**Keywords:** Water microbiology, Marine biology

## Abstract

Ciliate *Mesodinium* species are commonly distributed in diverse aquatic systems worldwide. Among *Mesodinium* species, *M. rubrum* is closely associated with microbial food webs and red tide formation and is known to acquire chloroplasts from its cryptophyte prey for use in photosynthesis. For these reasons, *Mesodinium* has long received much attention in terms of ecophysiology and chloroplast evolution. *Mesodinium* cells are easily identifiable from other organisms owing to their unique morphology comprising two hemispheres, but a clear distinction among species is difficult under a microscope. Recent taxonomic studies of *Mesodinium* have been conducted largely in parallel with molecular sequence analysis, and the results have shown that the best-known planktonic *M. rubrum* in fact comprises eight genetic clades of a *M. rubrum*/*M. major* complex. However, unlike the planktonic *Mesodinium* species, little is known of the genetic diversity of benthic *Mesodinium* species, and to our knowledge, the present study is the first to explore this. A total of ten genetic clades, including two clades composed of *M. chamaeleon* and *M. coatsi*, were found in marine sandy sediments, eight of which were clades newly discovered through this study. We report the updated phylogenetic relationship within the genus *Mesodinium* comprising heterotrophic/mixotrophic as well as planktonic/benthic species. Furthermore, we unveiled the wide variety of chloroplasts of benthic *Mesodinium*, which were related to the green cryptophyte *Chroomonas*/*Hemiselmis* and the red cryptophyte *Rhodomonas*/*Storeatula*/*Teleaulax* groups.

## Introduction

Ciliate *Mesodinium* species are commonly distributed in diverse aquatic environments and are either strictly heterotrophic or mixotrophic in their nutritional strategy^1–3^. *Mesodinium* species have a conspicuous morphology of the two hemispheres connected to two kinds of cirri (dikinetids and polykinetids), by which they can be easily distinguished from other ciliates. Meanwhile, their own morphological features (e.g., cirri and tentacles) make it difficult to distinguish among species within the genus because careful ultrastructural observations about them are required^4^. For this reason, recent classification of the genus *Mesodinium* has been increasingly based on the sequencing of nuclear genes (e.g., partial 18S rDNA and ITS rDNA) along with morphological observations^4–7^. Subsequently, some studies on the genetic diversity of *Mesodinium* in pelagic environments have shown that the mixotrophic *M. rubrum*, which has been recognized as the sole species causing massive nontoxic red tide blooms in estuaries and coasts around the world, actually comprise eight genetic variants of a *M. major*/*M. rubrum* species complex, indicating the existence of further diverse *Mesodinium* species in addition to *M. major* and *M. rubrum* in the pelagic environment^5,6^. *Mesodinium* is common in benthic environments as well, where four species (*M. chamaeleon*, *M. coatsi*, *M. pulex*, and *M. pupula*) have been reported^4,7–9^. However, little is known about the genetic diversity of *Mesodinium* in benthic ecosystems, unlike that of *Mesodinium* in pelagic conditions. The molecular approach to the species diversity of benthic *Mesodinium* is likely to reveal more diverse *Mesodinium* species in addition to the four benthic species already reported.

In particular, the mixotrophic *M. rubrum* is well known as a unique ciliate that acquires the chloroplast from its cryptophyte prey and utilizes it for photosynthesis; thus, this species is considered a model organism with clues to chloroplast evolution^10–12^. Some environmental studies on chloroplast diversity in *Mesodinium* have shown that the mixotrophic *M*. *rubrum*/*M. major* complex species retain prey chloroplasts originating from the *Teleaulax*/*Plagioselmis*/*Geminigera* (TPG) cryptophyte group, the most dominant type of which was *T. amphioxeia*^5,6,13,14^. Similar to *M*. *rubrum*/*M. major* complex species, benthic *M. chamaeleon* and *M. coatsi* were also able to retain chloroplasts sequestered from cryptophyte *Chroomonas* or *Rhodomonas* species and could sustain phototrophic growth by using some of these acquired chloroplast^9,15,16^. In addition, they seem to be accustomed to a wide range of cryptophyte chloroplasts, contrary to *M. rubrum* with chloroplast specificity to *T. amphioxeia*^17^. However, the type of chloroplasts retained in benthic *Mesodinium* species in natural environments has not yet been addressed.

In this study, we addressed the genetic diversity and chloroplast type of the benthic *Mesodinium* population from the sandy-sediment samples collected at different sites along the coastal beaches of Korea over several seasons. For identifications of *Mesodinium* and its chloroplast types, the nuclear partial 18S-entire ITS-partial 28S rDNA-specific primers for *Mesodinium* and chloroplast rbc*L* gene-specific primers for cryptophytes were used for PCR amplification. To obtain an ample amount of chloroplast gene information from the single-celled *Mesodinium*, the *rbc*L gene amplicons were especially sequenced using Illumina MiSeq sequencing platforms.

## Results

### Genetic diversity of benthic *Mesodinium*

**Figure 1:**
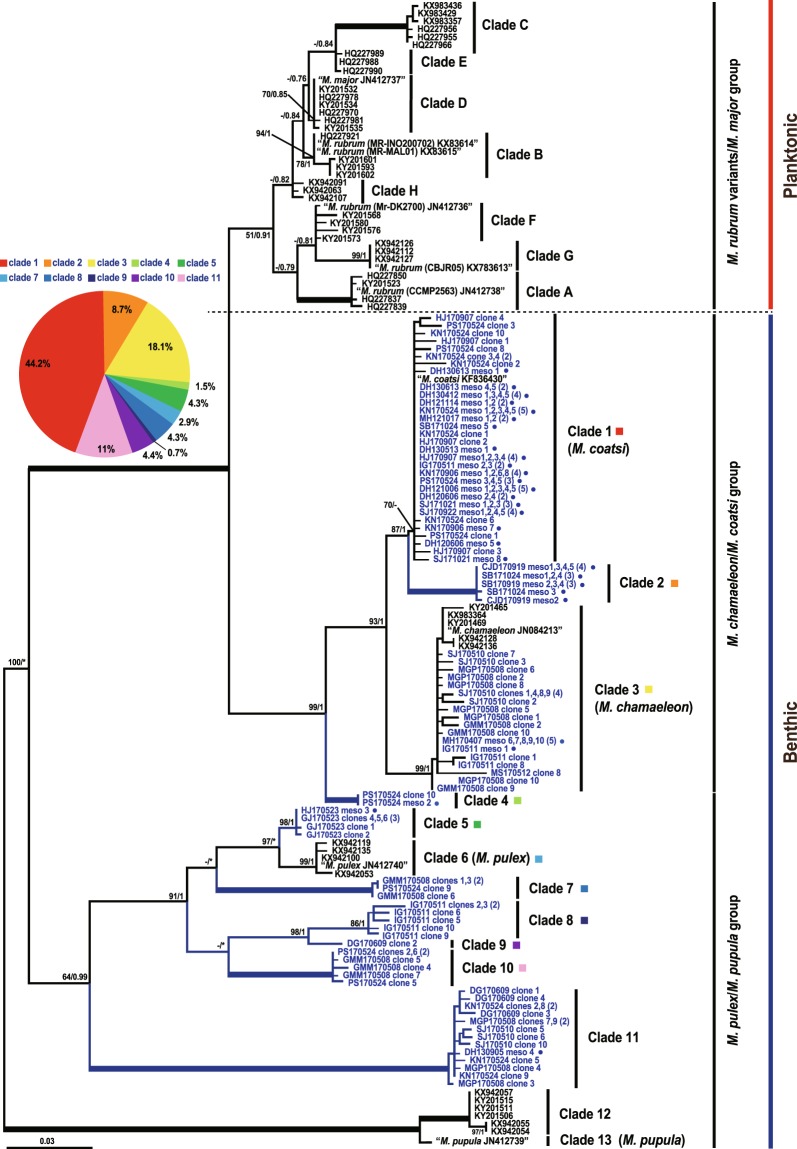
Consensus RAxML tree of *Mesodinium* species based on nuclear partial 18S-ITS-partial 28S rDNA gene. The maximum-likelihood bootstrap value (MLBT) and Bayesian posterior probability (PP) are shown at the branches. The bold branches denote strongly supported values of MLBT (100%) and PP (1.00). Hyphens indicate values less than 50% and asterisks represent the unmatched tree topology with the Bayesian tree. Letters and branches in blue denote sequences determined and clades newly generated in this study, respectively. A blue circle next to the letters represents a sequence retrieved from the single cell DNA. The pie chart shows the relative abundance of benthic *Mesodinium* clades which were detected in this study.

Our phylogeny considered the genetic relationship of all *Mesodinium* species in a wide range of aquatic environments, from the water column to sand sediment (Fig. 1). While planktonic *Mesodinium* populations were clustered into eight clades (clades A to H), the benthic *Mesodinium* populations were clustered into 13 clades from the 138 sequences obtained in this study, of which 69 were retrieved from 10 environmental samples by cloning and the remaining 69 from single cells by direct PCR analysis (Table 1). Of the 138 sequences obtained, 61 belonged to clade 1 with the largest ratio (44.2%), showing 98.3–100% sequence similarity with *M. coatsi*, and 25 belonged to clade 3 (18.1%), showing 97.3–100% similarity with *M. chamaeleon* (see the pie chart in Fig. 1).

**Table 1:** Description of the sampling locations and summary of the sequence analyses of the nuclear gene of *Mesodinium* species detected from single-cells and the environmental samples.

Location	Coordinate	Date	Abbrev	Sequences generated from		Benthic *Mesodinium* clades											
				Single cell DNA	Envir. DNA (No. of clones)	1	2	3	4	5	6	7	8	9	10	11	12
Mageompo	36°61′N 126°29′E	May-08-2017	MGP170508		10			6*								4*	
Sambong	36°56′N 126°31′E	Sep-19-2017	SB170919	3			3										
		Oct-24-2017	SB171024	5		1	4										
Chunjangdae	36°16′N 126°52′E	Sep-19-2017	CJD170919	5			5										
Mohang	35°58′N 126°50′E	Oct-17-2012	MH121017	2		2											
		Apr-07-2017	MH170407	5				5									
Dongho	35°51′N 126°48′E	Jun-06-2012	DH120606	3		3											
		Oct-06-2012	DH121006	5		5											
		Nov-14-2012	DH121114	2		2											
		Apr-12-2013	DH130412	4		4											
		May-13-2013	DH130513	1		1											
		Jun-13-2013	DH130613	3		3											
		Sep-05-2013	DH130905	1												1	
Gamami	35°58′N 126°50′E	May-08-2017	GMM170508		9			3*				3*			3*		
Ikgeum	34°25′N 127°08′E	May-11-2017	IG170511	3	8	2		1/2*					6*				
Songjeong	34°43′N 128°01′E	May-10-2017	SJ170510		10			7*								3*	
		Sep-22-2017	SJ170922	4		4											
		Oct-21-2017	SJ171021	4		4											
Dogu	35°59′N 129°27′E	Jun-09-2017	DG170609		4									1*		3*	
Mangsang	37°35′N 129°05′E	May-12-2017	MS170512		1			1*									
Hyeopjae	33°23′N 126°14′E	May-23-2017	HJ170523	1						1							
		Sep-07-2017	HJ170907	4	4	4/4*											
Gwakji	33°27′N 126°18′E	May-23-2017	GJ170523		5					5*							
Kimnyeong	33°33′N 126°45′E	May-24-2017	KN170524	5	10	5/6*										4*	
		Sep-06-2017	KN170906	5		5											
Pyoseon	33°19′N 126°50′E	May-24-2017	PS170524	4	8	3/3*			1/1*			1*			3*		
**Total Seq. No.**				69	69												
					**138**	61	12	25	2	6	0	4	6	1	6	15	0

The asterisk means the sequence number from the environmental sample.

The other sequences generated eight new clades (2, 4, 5, 7, 8, 9, 10, and 11). The 12 sequences retrieved from the 12 single cells isolated from two different sites (Sambong and Chunjangdae) clustered together to form clade 2, the phylogenetic position of which was between clade 1 (*M*. coatsi) and clade 3 (*M. chamaeleon*). Only two sequences, ‘PS170524 clone 10’ and ‘PS170524 meso 2’, retrieved from the environmental samples and a single cell, respectively, formed the new clade 4 with strong node values (bootstrap 99%, posterior probability 1). Clade 4 was close to clades 1, 2, and 3 but branched at the base of these clades. Clade 5, which comprised a single cell sequence ‘HJ170523 meso 3’ and 5 environmental clones of ‘GJ170523’, showed a sister group relationship to clade 6 (*M. pulex*). Clades 7, 8, 9, and 10 comprised the clone libraries without single cell sequences. Clade 9 comprised only one environmental clone sequence ‘DG170609 1’. Clade 11, which was broadly positioned between heterotrophic *M. pupula* and *M. pulex* clades, comprised 14 environmental clones and a single cell sequence. Meanwhile, none of the clones analyzed in this study was included in clade 6 (*M. pulex*), clade 12, and clade 13 (*M. pupula*).

**Figure 2:**
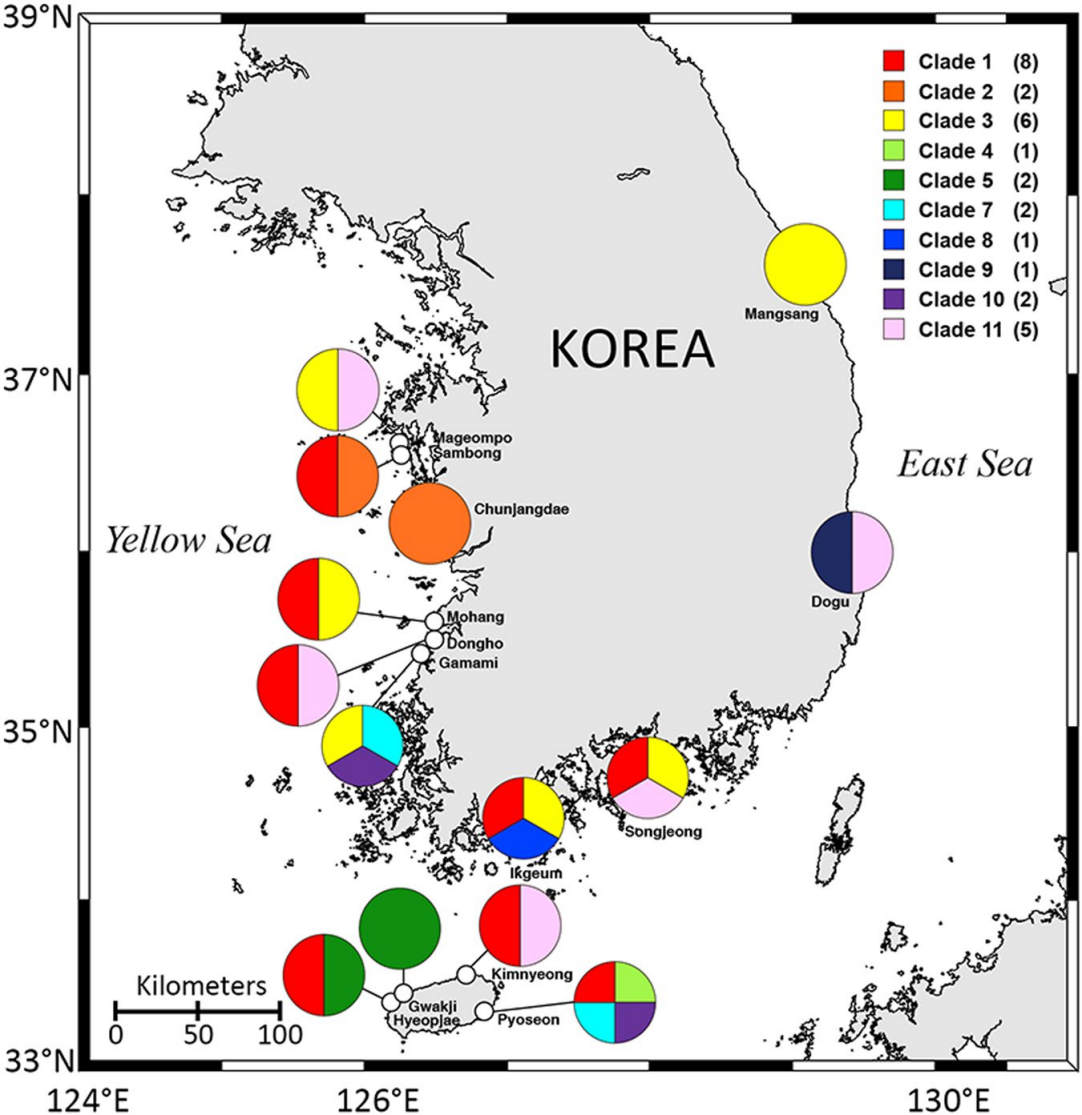
Geographic diversity of benthic *Mesodinium* species discovered from coastal beaches of Korea. Colors represent each genetic clade for benthic *Mesodinium*. The number in parentheses refers to the number of locations where each clade was found.

Clade 1 (*M. coatsi*) was the most frequent in 8 of 14 sites, followed by clade 3 (*M. chamaeleon*) in 6 sites, and clade 11 in 5 sites (Fig. 2). Clades 2, 5, 7, and 10 were established by sequence data retrieved from at least two sites, while clades 4, 8, and 9 were generated by the sequences obtained from only one site.

### Screening for chloroplast *rbc*L gene in natural benthic *Mesodinium* population

**Figure 3:**
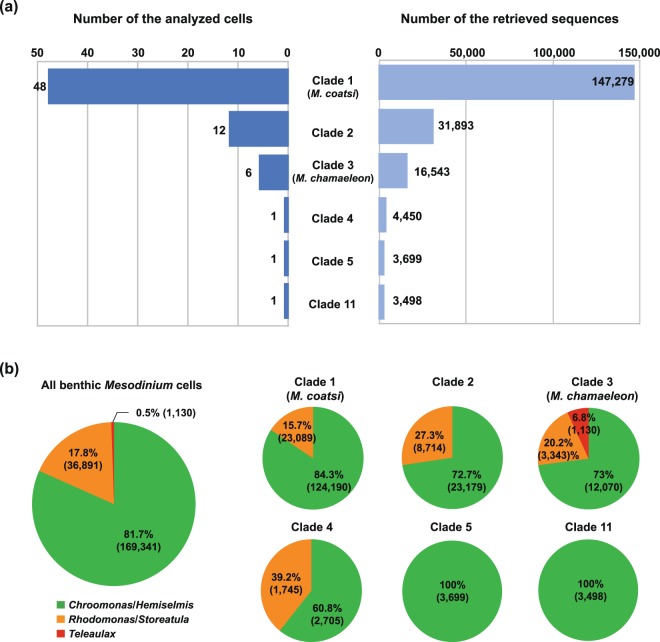
(**a**) Number of cells analyzed for chloroplast diversity using MiSeq platform (left) and the number of chloroplast sequences (*rbc*L gene) retrieved (right). (**b**) Relative abundance of chloroplast type to all analyzed *Mesodinium* cells (a large circle diagram) and to each clade (six small circle diagram).

**Figure 4:**
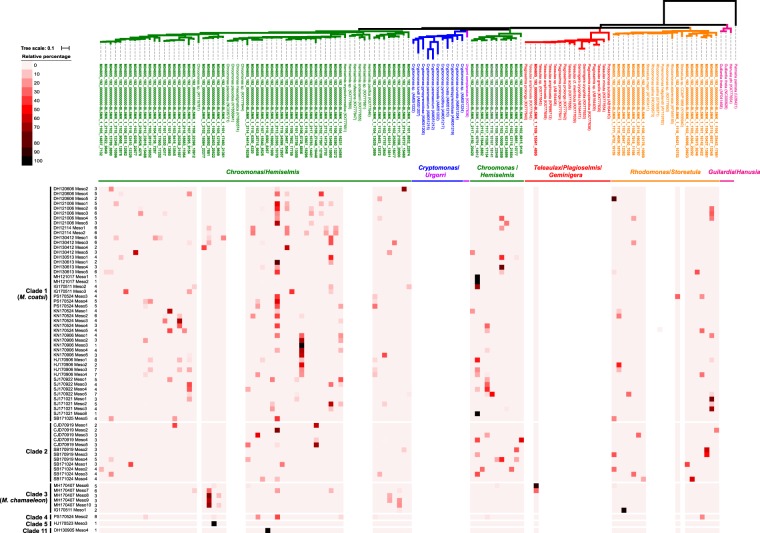
Heat map with RAxML tree of dominant OTUs (*rbc*L gene sequences) within benthic *Mesodinium* cells from coastal beaches of Korea. The upper phylogeny was constructed based on chloroplast *rbc*L gene sequences of cryptophytes at the top of the heatmap. Colored branches indicate different cryptophyte genera. Color ranges for the heatmap represent the relative percentage of each OTU in each cell (upper left). The number on the left side of the heatmap means total number of detected OTUs in a single cell.

A total of 207,362 sequences of the chloroplast *rbc*L gene were obtained from 69 individual *Mesodinium* cells, which were recovered mostly from 40 *M. coatsi*-like single cells (Fig. 3a). These sequences were 88–100% similar to the sequences of five cryptophyte genera (*Chroomonas*, *Hemiselmis*, *Rhodomonas*, *Storeatula*, and *Teleaulax*) and clustered into 80 operational taxonomic units (OTUs) (see upper phylogeny in Fig. 4). Among the 80 OTUs, 64 were affiliated with the green cryptophyte *Chroomonas*/*Hemiselmis* group, accounting for about 81.7% (169,341 sequences) of the total retrieved sequences (Fig. 3b). The other 15 OTUs (17.8%; 36,891 sequences) were associated with the red cryptophyte *Rhodomonas*/*Storeatula* group. The remaining 1 OTU (0.5%; 1,130 sequences) was associated with the red cryptophyte *Teleaulax*, which was found in only two cells belonging to clade 3 (MH 170407 meso6 and MH 170407 meso7) (Fig. 4). All benthic *Mesodinium* cells always contained green chloroplasts, approximately half of which (46.4%) had red chloroplasts in addition to the green ones (Fig. 4). The dominance of the green chloroplasts was evident even when analyzed by each clade (Fig. 3b).

Proportional abundance of each chloroplast OTU in a single cell was represented as a heat map using a color gradient from black (100%) to pale red (0%) (Fig. 4). Most of the 69 *Mesodinium* cells (63 cells, 91.3%) contained more than two types of chloroplasts simultaneously in a single cell, with a maximum of 8 types of chloroplasts retained. Only six cells belonging to clades 1, 5, and 11 had only one type of chloroplast.

## Discussion

While the genetic diversity and chloroplast screening of *Mesodinium* have been mostly carried out in pelagic ecosystems^5,6^, those of the *Mesodinium* species inhabiting benthic environments have not been explored at all. To our knowledge, this is the first study to show the large genetic diversity and perform chloroplast identification of benthic *Mesodinium*.

The genetic diversity of *Mesodinium* in the benthic ecosystem was much more varied than that of *Mesodinium* in the pelagic domain. Phylogeny based on the partial nuclear SSU-ITS region-partial LSU rDNA showed that planktonic *Mesodinium* comprised 8 clades of the *M. major*/*M. rubrum* complex, whereas benthic *Mesodinium* comprised 13 genetic clades, 10 of which were newly detected in this study. In detail, two of the 10 clades were benthic *M. coatsi* (clade 1) and *M. chamaeleon* (clade 3), and the other eight clades (2, 4, 5, 7, 8, 9, 10, and 11) were new ones discovered through this study. Presently, among the 13 genetic clades, except for four clades (1, 3, 6, and 13) of the benthic *Mesodinium* species (*M. coatsi*, *M. chamaeleon*, *M. pulex*, and *M. pupula*, respectively), nine clades (2, 4, 5, 7, 8, 9, 10, 11, and 12) are genetically different groups from the four species clades. Further, when considering the genetic distances among clades, all unnamed clades are thought to be new *Mesodinium* species because the distances among the new clades are much larger than those between the benthic *M. coatsi* (clade 1) and *M. chamaeleon* (clade 3), and particularly those between planktonic *M. major* (clade D) and *M. rubrum* (clade F), whose morphological characteristics were already proven to be different from each other. In fact, *M. major* (JN412737), which had been previously considered as one of the *M. rubrum* variants^5^, was described as a new species^4^. In this context, some planktonic clades (C, D, E, G, and H) having marked genetic differences from *M. major* and *M. rubrum*, but collectively called as the *M. major*/*M. rubrum* complex, are also likely to be new species^5,6^. The present study greatly expanded the genetic diversity information of the ciliate genus *Mesodinium*, bringing the total up to at least 21 genetic clades across pelagic and benthic environments. This also indicates that the more we explore a variety of environments, the more diverse new clades (i.e., species) could be discovered. Indeed, a new *Mesodinium* sequence that was phylogenetically positioned between the planktonic *M. rubrum*/*M. major* group and the benthic *M. chamaeleon*/*M. coatsi* group has recently been reported from a brackish lake^18^.

The phylogeny of *Mesodinium* could be largely divided into three groups based on their nutrition strategy: *M. rubrum*/*M. major* group, *M. chamaeleon*/*M. coatsi* group, and *M. pulex*/*M. pupula* group. The first two groups are mixotrophic species and are known to perform temporary photosynthesis by using stolen chloroplasts from cryptophyte prey^15,16,19^. Nonetheless, *M. rubrum*/*M. major* group is much closer to being phototrophic^11,12,20,21^. In contrast, the third group is a heterotrophic species feeding on diverse prey organisms, including cryptophytes and a dinoflagellate^22,23^. Given the phylogenetic positions of these three groups with different nutritional modes, clades 2 and 4 included in the mixotrophic *M. chamaeleon*/*M. coatsi* group are likely to be new kleptoplastidic ciliates, and clades 6, 7, 8, 9, 10, 11, and 12 belonging to the *M. pulex*/*M. pupula* groups are assumed to be new heterotrophic ciliates.

During the study period, mixotrophic *M. coatsi* (clade 1) was the most common in many sampling sites, followed by mixotrophic *M. chamaeleon* (clade 3) which is a sister group to *M. coatsi*. Notably, their temporal occurrence patterns were distinctly different during the study period. While *M. coatsi* frequently occurred throughout the year, *M. chamaeleon* was found only in April and May. Contrary to the result from the present study, however, Johnson *et al*.^6^ reported that *M. chamaeleon*-like species were the most commonly present at three global locations (Baltic Sea and south and north Pacific regions) in different seasons, but *M. coatsi*-type species were not found at all. Perhaps, the interpretation of these apparently conflicting results should take into account the location of the environmental samplings: while Johnson’s group collected *Mesodinium* samples from surface waters, we collected samples from sandy sediments. Alternatively, the occurrence of the two species may vary depending on the environmental conditions (including the composition and abundance of prey), seasonal changes, and geographical locations.

As mentioned above, the benthic species *M. chamaeleon*, *M. pulex* and *M. pupula* were found worldwide even in the surface seawaters^6^. The *Mesodinium* species, with a tendency of motility falling down to the bottom (e.g., *M. chamaeleon*, *M. coatsi*, and *M. pupula*), have been mostly found in sediment interstitial waters or shallow water above the sand surface^4,7^. However, considering that *M. pulex* and *M. pupula* are able to inhabit the water column as well as bottom environments^4,24–27^, benthic *Mesodinium* species are expected to also be observed in the water column because of resuspension from the bottom into the upper layer owing to high turbulence.

Previous field observation has shown that *M. rubrum* has chloroplast specificity, mostly toward the cryptophyte *T. amphioxeia*^14^. Laboratory experiments have also shown that *M. rubrum*, in particular, prefers *Teleaulax*-like species over other cryptophyte prey^17^. By comparison, benthic *Mesodinium* species in this study were able to retain a large variety of cryptophyte chloroplasts belonging to *Chroomonas*/*Hemiselmis*/*Rhodomonas*/*Storeatula*, and *Teleaulax*, with a total of 80 types being detected. Such chloroplast diversity is considered a response to the feeding of all cryptophytes randomly encountered in the field. Indeed, all mixotrophic *M. rubrum*, *M. chamaeleon*, and *M. coatsi* were able to ingest all provided cryptophyte prey regardless of whether the prey organisms supported their sustained growth^15–17^.

Furthermore, most benthic *Mesodinium* cells (91.3%) were observed to retain multiple types of the chloroplasts (up to eight) simultaneously, which is assumed to be a cumulative result of chloroplasts retained for photosynthesis. Not all retained chloroplasts, of course, might be photosynthetically active, and some could be detected as aged chloroplasts being digested or as the simple uptake of prey with little or no photosynthetic performance. For example, only two cells, designated here as HJ 170523 meso3 and DH 130905 meso4 (belonging to the clades 5 and 11, respectively), which are included in the heterotrophic *M*. *pulex*/*M*. *pupula* groups, were found to have one type of chloroplast. Given that the two cells are heterotrophs based on their phylogenetic position, the detection of one kind of chloroplast may be the result of digestion in progress rather than it being used for photosynthesis.

When we isolated benthic *Mesodinium* species with chloroplasts under an inverted microscope, most of them had green chloroplasts. However, unexpectedly, approximately half of the *Mesodinium* cells simultaneously had both green chloroplasts (phycocyanin) and also red chloroplasts (phycoerythrin) within a single cell. Previous field observations have shown that benthic *Mesodinium* species commonly retained green rather than red chloroplasts or both^4,9,15^. Perhaps the early observations overlooked the red chloroplasts that existed in small quantities or were embedded within green chloroplasts. Nonetheless, the benthic *Mesodinium* species predominantly possessed green rather than red chloroplasts. Furthermore, it is of interest to note that the green chloroplasts originating from *Chroomonas*/*Hemiselmis* were being retained at all times in all cells. Even in the cells with both chloroplasts, the green ones were relatively more abundant than the red ones, which were derived from *Rhodomonas*/*Storeatula* and/or *Teleaulax*. Why are the green rather than the red chloroplasts remarkably predominant within benthic *Mesodinium* species? The abundance and diversity of benthic cryptophytes seem to be closely reflected in the chloroplast diversity of benthic *Mesodinium*, which suggests that the green cryptophytes *Chroomonas* and *Hemiselmis* would generally be much more abundant than were the red cryptophytes *Rhodomonas* and *Storeatula*. We have indeed observed that in field samples green cryptophytes usually appear in more abundance than did red cryptophytes (personal observation). As in benthic species, coexistence of green and red chloroplasts was occasionally observed in planktonic and brackish *Mesodinium* species^18,28^. However, contrary to *Mesodinium* species in the benthic environment, the red chloroplasts associated with the TPG group are predominant in planktonic *Mesodinium* species^5,14^. Indeed, TPG cryptophytes commonly appeared in marine water columns, where *M. rubrum* bloomed^6^. In a brackish lake, meanwhile, the chloroplasts in the *Mesodinium* shifted seasonally from red chloroplasts (*T*. *amphioxeia*) to green chloroplasts (*Hemiselmis* sp.) during the occurrence of ecological succession from *Teleaulax* to *Hemiselmis*^18^. Taken together, the dominant chloroplast type within *Mesodinium* species seems to depend upon cryptophyte availability in their habitat.

We have proven that the red chloroplasts in natural benthic *Mesodinium* cells were exclusively from the *Rhodomonas*/*Storeatula* group. In contrast, the red chloroplasts of planktonic *Mesodinium* cells have been affiliated with TPG cryptophytes, suggesting that the TPG group in the pelagic domain and *Rhodomonas*/*Storeatula* group in the benthic domain are the predominant red cryptophytes. Nonetheless, some *T. amphioxeia* chloroplasts were surprisingly detected from only two *M. chamaeleon*-like cells (MH 170407 meso6 and MH170407 meso7). It does not seem to be the unique feeding characteristic of *M. chamaeleon* alone. Rather, given that the sampling sites were in the intertidal zone with semidiurnal cycles, it is likely that the cells resuspended by tidal mixing may have ingested the cryptophyte *T. amphioxeia* in the water column and/or vice versa.

In conclusion, our study on genetic diversity of benthic *Mesodinium* species unveiled the presence of more diversity in the ciliate *Mesodinium* than that previously thought, bringing the total to at least 21 genetic clades across pelagic and benthic environments, although morphological characterizations are still necessary. In addition, the chloroplasts diversity of benthic *Mesodinium* suggests that benthic/mixotrophic *Mesodinium* species, phylogenetically positioned between the mixotrophic *M. rubrum*/*M. major* complex group and the heterotrophic *M. pulex*/*M. pupula* group, are highly diverse and seem to be at the intermediate nutrition mode between heterotrophy and mixotrophy.

## Materials and Methods

### Sample collection and DNA extraction

Surface sediment samples were collected at low tide using a flat spoon from ten different sites along the coastal beaches of Korea from June 2012 to October 2017 (Table 1). Samples, which were filled with interstitial sediment seawater, were stored in zipper bags and taken to the laboratory. Sediment samples with seawater were poured into an open plastic cylinder (300 × 90 nm diameter) with a 60 μm mesh at the bottom. For 1 h, seawater flowing through the sediment was collected in a petri dish (100 × 40 mm, SPL, Korea) placed underneath the cylinder^29^. From these seawater samples, environmental DNA and single *Mesodinium* cell DNA were obtained by different methods. For environmental DNA extraction, the seawater samples were filtered through a 0.2 μm pore size Supor filter (47 mm, PALL, USA) and the filters were stored in a 5 ml snap cap tube (SPL, Korea) containing 2 ml STE buffer (100 mM NaCl, 10 mM Tris-HCl, 1 mM EDTA, pH 8.0) at 80 °C until the next step. Prior to the extraction of genomic DNA, the filters were first cut into small pieces^5^. DNA was extracted according to the protocol presented in previous studies^30,31^ with some modifications. For the single-cell DNA extraction, individual *Mesodinium* cells isolated from each sediment sample were put into separate 0.2 ml PCR tubes containing 50 μL of 10% Chelex (Bio-Rad, USA). The PCR tubes were boiled at 95 °C for 1 h and then centrifuged at 13,000 rpm for 5 min. Chelex supernatant (30 μL) was withdrawn as a template for PCR.

### Amplification of nuclear partial 18S-ITS-partial 28S rDNA and sequencing

DNAs extracted from the environmental samples and single cells were subjected to PCR amplification of the nuclear partial 18S-entire ITS- partial 28S rDNA for benthic *Mesodinium* using the *Mesodinium*-specific primers^6^, MESO1200F and MESO28_S (Table 2). The PCR was performed in a 50 μl volume containing 5 μl 10X Taq polymerase buffer, 0.2 mM dNTP, 0.1 μM primers (MESO1200F and MESO28S_R), and 1.25 units of Diastar-Taq DNA polymerase (Solgent, Korea) on a C1000 Touch thermal cycler (Bio-Rad, USA). Reactions were run following the PCR program: initial denaturation at 95 °C for 5 min; 40 cycles of 95 °C for 1 min, 55 °C for 1 min, and 72 °C for 1 min 30 s, and final extension at 72 °C for 10 min. Only in some samples with insufficient PCR product, was a semi-nested PCR required to obtain enough DNA for subsequent cloning using a second pair of primers, MESO1440F and MESO28S_R (approximately 715 bp). In the nested PCR reactions, 5 μl of PCR product from the first PCR round was used as a template and PCR conditions were run as above, except for 25 instead of 40 cycles. The amplified gene fragments were purified using a PCR purification kit (Bioneer, Korea) and ligated into the pGEM-T Easy vector supplied with the pGEM-T Easy Vector System (Promega, USA) according to the manufacture’s protocols. Plasmid DNA from putative positive colonies were harvested using a PCR purification kit (Bioneer, Korea). Four to then positive clones from each sampling site were sequenced using the T7 and SP6 promoter primers by Sanger sequencing (Cosmo Genetech, Korea).

**Table 2:** Primers used in this study. Illumina forward and reverse overhang adaptors were attached to the primers for *rbc*L gene.

Primer			Remarks
**Nuclear partial SSU-entire ITS-partial LSU for** ***Mesodinium*** **species**			
	**Sequence**		
MESO1200F	ATTCCGGTAACGAACGAGAC		Johnson *et al*.^6^
MESO1440F	AACTAGGAATGTCTCGTAAGC		Johnson *et al*.^6^
MESO28SR	AGACTTGGATGACTTTTATCACC		Johnson *et al*.^6^
**Chloroplast** ***rbc*** **L gene for Cryptophytes**			
	**Overhang adaptor**	**Target sequence**	
CryprbcL646F	TCGTCGGCAGCGTCAGATGTGTATAAGAGACAG	ATGAGATGGARAGAGCGTTTC	This study
Crypt_rbcLR2	GTCTCGTGGGCTCGGAGATGTGTATAAGAGACAG	CAGTGRATACCACCWGAAGCWA	Modified from Johnson *et al*.^6^

### Amplification of chloroplast *rbc*L gene, sequencing, and data analyses

To elucidate the genetic diversity of chloroplasts within benthic *Mesodinium* cells, high-throughput sequencing was performed using an MiSeq platform following the manual described at the Illumina website (http://support.illumina.com/ downloads/16s_metagenomic_sequencing_library_preparation.html). The DNAs obtained from a total of 69 single cells were used as a template to amplify the chloroplast *rbc*L gene. The cryptophyte-specific primers, newly designed CryprbcL646F and modified Cryp_rbcLR2^6^, equipped with Illumina adapter sequences were used for the first round PCR (Table 2). The PCR condition was 95 °C for 5 min; 35 cycles of 95 °C for 1 min, 50 °C for 30 s, and 72 °C for 1 min; and a final extension at 72 °C for 10 min. The second round PCR for attaching Illumina barcodes was carried out using the index primers. Each of the dual indexed samples was purified and then quantified using a Qubit 3.0 Fluorometer (Thermo Fisher Scientific, Waltham, USA). An equal amount of each DNA was pooled and then sequenced by the Illumina MiSeq2 × 300 PE platform (ChunLab, Seoul, Korea). The obtained sequence data was processed using Mothur^32^. The paired-end sequences were assembled and aligned. Low quality sequences including erroneous lengths (>525 bp) and homopolymers (>8) were removed. Taxonomic assignment of the sequences was based on the aligned reference database of all available cryptophyte *rbc*L genes registered in GenBank. The data matrix processed by the Mothur was manipulated for further analysis manually in an Excel worksheet. The OTUs with either single reads or sequences with nonsense codons were removed. The relative abundance of OTU was ranked for the observed OTUs number in different cells, excluding the OTUs less than 1% of the total number, and summarized for each cell or group in a heatmap and a circle diagram, respectively.

### Phylogenetic analysis

Nuclear and chloroplast sequences were assembled using ContigExpress and trimmed to ensure that all had the same start and end points. All clone sequences were checked for PCR artifacts and chimeras using DECIPHER^33^ and six potential chimera sequences were excluded from analysis. The alignment data comprised 196 *Mesodinium* partial 18S-ITS- partial 28S rRNA gene sequences obtained from GenBank, environmental clone libraries, and the single cell sequences. Of the environmental clone sequences, the six identified as chimeric sequences were excluded from the alignment data. Alignment was constructed by eye using Genetic Data Environment (GDE 2.4) and positions which could not be aligned unambiguously were omitted from analysis. Maximum likelihood phylogeny was analyzed using RAxML version 8, with a GTR + GAMMA model obtained automatically. Bootstrap values were calculated using 2,000 replicates with the same substitution model. For Bayesian analyses, a likelihood-ratio test was performed using Modeltest (version 3.7) to determine the best model for combined data set. Bayesian analyses were run with the MCMC process for 20,000,000 generations, retaining one tree in every 1,000 generations and the first 8,000 trees were discarded. Trees were visualized using Figtree version 1.4.2. A heat map with the maximum likelihood tree of the chloroplast *rbc*L gene was visualized using the iTOL webtool^34^.

## Supplementary information

Table S1

## Data Availability

All sequences of the partial 18S-ITS-partial 28S rDNA obtained from this study are deposited in GenBank under the accession numbers MN059710-MN059847. The raw sequence data are deposited in the NCBI Sequence Read Archive (SRA) database with the BioSample accession ID SAMN12027021 under the BioProject PRJNA548425 (http://www.ncbi.nlm.nih.gov/biosample).
