# Supplementary material for: Unveiling the hidden genetic diversity and chloroplast type of marine benthic ciliate Mesodinium species
Source: Sci Rep. 2019 Oct 1;9:14081. doi: 10.1038/s41598-019-50659-2 (PMC6773952; doi:10.1038/s41598-019-50659-2)
Supplement: Supplementary file 1 — Table S1 [file 41598_2019_50659_MOESM1_ESM.pdf]

Supplementary information

Unveiling the hidden genetic diversity and chloroplast type of marine benthic ciliate *Mesodinium* species

Miran Kim[1] **& Myung Gil Park**[2*]

[1]Research Institute for Basic Science, Chonnam National University, Gwangju 61186, Republic of Korea

[2]LOHABE, Department of Oceanography, Chonnam National University, Gwangju 61186, Republic of Korea

Correspondence author: Myung Gil Park (email: mpark@chonnam.ac.kr)

Supplementary Table S1. Genbank accession numbers for sequences of the partial 18S, entire ITS, and partial 28S rDNA genes of benthic *Mesodinium* cells and/or environmental samples used in this study.

Accession number	Sequence name	source
MN059710	KN170524clone1	environmetal sample
MN059711	KN170524clone10	environmetal sample
MN059712	KN170524clone3	environmetal sample
MN059713	KN170524clone4	environmetal sample
MN059714	KN170524clone6	environmetal sample
MN059715	KN170524clone7	environmetal sample
MN059716	PS170524clone3	environmetal sample
MN059717	PS170524clone8	environmetal sample
MN059718	PS170524clone1	environmetal sample
MN059719	HJ170907clone1	environmetal sample
MN059720	HJ170907clone2	environmetal sample
MN059721	HJ170907clone3	environmetal sample
MN059722	HJ170907clone4	environmetal sample
MN059723	IG170511meso3	single cell
MN059724	DH130613meso5	single cell
MN059725	DH130613meso4	single cell
MN059726	DH130613meso1	single cell
MN059727	DH130513meso1	single cell
MN059728	DH130412meso5	single cell
MN059729	DH130412meso4	single cell
MN059730	DH130412meso3	single cell
MN059731	IG170511meso2	single cell
MN059732	DH130412meso1	single cell
MN059733	DH121114meso2	single cell
MN059734	DH121114meso1	single cell
MN059735	MH121017meso2	single cell
MN059736	MH121017meso1	single cell
MN059737	DH121006meso5	single cell
MN059738	DH121006meso4	single cell
MN059739	DH121006meso3	single cell
MN059740	DH121006meso2	single cell
MN059741	DH121006meso1	single cell
MN059742	DH120606meso5	single cell
MN059743	DH120606meso4	single cell
MN059744	DH120606meso2	single cell
MN059745	SB171024meso5	single cell
MN059746	SJ171021meso8	single cell
MN059747	SJ171021meso3	single cell
MN059748	SJ171021meso2	single cell
MN059749	SJ171021meso1	single cell
MN059750	SJ170922meso5	single cell
MN059751	SJ170922meso4	single cell
MN059752	SJ170922meso2	single cell
MN059753	SJ170922meso1	single cell
MN059754	HJ170906meso4	single cell
MN059755	HJ170906meso3	single cell
MN059756	HJ170906meso2	single cell
MN059757	HJ170906meso1	single cell
MN059758	KN170906meso8	single cell
MN059759	KN170906meso7	single cell
MN059760	KN170906meso6	single cell
MN059761	KN170906meso2	single cell
MN059762	KN170906meso1	single cell
MN059763	KN170524meso5	single cell
MN059764	KN170524meso4	single cell
MN059765	KN170524meso3	single cell
MN059766	KN170524meso2	single cell

MN059767	KN170524meso1	single cell
MN059768	PS170524meso5	single cell
MN059769	PS170524meso4	single cell
MN059770	PS170524meso3	single cell
MN059771	SB171024meso4	single cell
MN059772	SB171024meso3	single cell
MN059773	SB171024meso2	single cell
MN059774	SB171024meso1	single cell
MN059775	KJP170919meso4	single cell
MN059776	KJP170919meso3	single cell
MN059777	KJP170919meso2	single cell
MN059778	CJD170919meso5	single cell
MN059779	CJD170919meso4	single cell
MN059780	CJD170919meso3	single cell
MN059781	CJD170919meso2	single cell
MN059782	CJD170919meso1	single cell
MN059783	SJ170510clone1	environmetal sample
MN059784	SJ170510clone2	environmetal sample
MN059785	SJ170510clone3	environmetal sample
MN059786	SJ170510clone4	environmetal sample
MN059787	SJ170510clone8	environmetal sample
MN059788	SJ170510clone7	environmetal sample
MN059789	SJ170510clone9	environmetal sample
MN059790	IG170511clone1	environmetal sample
MN059791	IG170511clone8	environmetal sample
MN059792	GMM170508clone2	environmetal sample
MN059793	GMM170508clone10	environmetal sample
MN059794	GMM170508clone9	environmetal sample
MN059795	MGP170508clone10	environmetal sample
MN059796	MGP170508clone1	environmetal sample
MN059797	MGP170508clone2	environmetal sample
MN059798	MGP170508clone5	environmetal sample
MN059799	MGP170508clone6	environmetal sample
MN059800	MGP170508clone8	environmetal sample
MN059801	MS170512clone8	environmetal sample
MN059802	IG170511meso1	single cell
MN059803	MH170407meso10	single cell
MN059804	MH170407meso9	single cell
MN059805	MH170407meso8	single cell
MN059806	MH170407meso7	single cell
MN059807	MH170407meso6	single cell
MN059808	PS170524clone10	environmetal sample
MN059809	PS170524meso2	single cell
MN059810	GJ170523clone1	environmetal sample
MN059811	GJ170523clone2	environmetal sample
MN059812	GJ170523clone4	environmetal sample
MN059813	GJ170523clone5	environmetal sample
MN059814	GJ170523clone6	environmetal sample
MN059815	HJ170523meso3	single cell
MN059816	GMM170508clone1	environmetal sample
MN059817	GMM170508clone3	environmetal sample
MN059818	GMM170508clone6	environmetal sample
MN059819	PS170524clone9	environmetal sample
MN059820	IG170511clone9	environmetal sample
MN059821	IG170511clone5	environmetal sample
MN059822	IG170511clone6	environmetal sample
MN059823	IG170511clone2	environmetal sample
MN059824	IG170511clone3	environmetal sample
MN059825	IG170511clone10	environmetal sample
MN059826	DG170906clone2	environmetal sample
MN059827	GMM170508clone5	environmetal sample

MN059828	GMM170508clone4	environmetal sample
MN059829	GMM170508clone7	environmetal sample
MN059830	PS170524clone2	environmetal sample
MN059831	PS170524clone5	environmetal sample
MN059832	PS170524clone6	environmetal sample
MN059833	DH130905meso4	single cell
MN059834	SJ170510clone6	environmetal sample
MN059835	SJ170510clone5	environmetal sample
MN059836	SJ170510clone10	environmetal sample
MN059837	KN170524clone2	environmetal sample
MN059838	KN180524clone8	environmetal sample
MN059839	KN170524clone9	environmetal sample
MN059840	KN170524clone5	environmetal sample
MN059841	MGP170508clone3	environmetal sample
MN059842	MGP170508clone4	environmetal sample
MN059843	MGP170508clone7	environmetal sample
MN059844	MGP170508clone9	environmetal sample
MN059845	DG170906clone1	environmetal sample
MN059846	DG170906clone3	environmetal sample
MN059847	DG170906clone4	environmetal sample
